# Lavender aromatherapy: A systematic review from essential oil quality and administration methods to cognitive enhancing effects

**DOI:** 10.1111/aphw.12310

**Published:** 2021-10-05

**Authors:** Eleonora Malloggi, Danilo Menicucci, Valentina Cesari, Sergio Frumento, Angelo Gemignani, Alessandra Bertoli

**Affiliations:** ^1^ Department of Surgical, Medical and Molecular Pathology and Critical Care Medicine University of Pisa Pisa Italy; ^2^ Department of Pharmacy University of Pisa Pisa Italy

**Keywords:** arousal, attention, cognitive functions, inhalation, lavender, memory

## Abstract

Modern society is reviving the practice of aromatherapy, and lavender is reported being the most worldwide purchased plant for essential oil (EO) extraction. Since recent studies reported cognitive enhancing effects of lavender besides the hypno‐inducing effects, a literature review is needed. Considering EO quality and diffusion devices, we conducted a systematic review on the effects of lavender EO inhalation on arousal, attention and memory in healthy subjects. Starting from this new multidisciplinary perspective, cognitive effects were reviewed to link outcomes to effective and reproducible protocols. A systematic search on MEDLINE, ERIC, PsycInfo, Google Scholar, and Scopus databases using Cognitive Atlas and plant‐related keywords was conducted. Among the 1,203 articles yielded, 11 met eligibility criteria. Subjects administered with lavender EO displayed arousal decrease and sustained attention increase. Controversial results emerged regarding memory. Lack of EO quality assessment and protocols heterogeneity did not allow assessing whether different EO composition differentially modulates cognition and whether placebo effect can be discerned from EO effect itself. However, GABAergic pathway modulation exerted by linalool, a major lavender EO constituent, might explain cognitive functions empowerment. We speculate aromatherapy could be a burgeoning cognition enhancing tool, although further investigation is required to reach robust conclusions.

**Abbreviation**
EO: essential oils

## INTRODUCTION

Over the last decade, scientific literature revived the traditional medical practice of aromatherapy, defined as “the controlled use of plant essences for therapeutic purposes” (Ernst, 2000; Hoffmann, 2003), and contributed to make it rise to the role of the most frequently used complementary medicine.

Indeed, the International Federation of Essential Oils and Aroma Trades (IFEAT) reported a rapid growth in aromatic plants cultivation and lavender as the most worldwide purchased raw material for essential oils (EO) extraction. EO are a complex mixture of chemical volatile components obtained by different extraction techniques (distillation or cold pressure) avoiding any sort of chemical solvent (Petersen, 2013). Currently, since the fortuitous discovery of lavender's soothing effects in the early 1900s by the French chemist Gattefossé (Miller & Miller, 1999), lavender aromatherapy has widely spread among alternative or complementary usages, ranging from antiemetic to soothing and pain treatment up to lately discovered sedative and cognitive effects. Indeed, Gattefossé began to study aromatherapy and inspired the physician Jean Valnet to lay the foundations of plant‐based medicine, by implementing lavender EO to soothe the injuries of the veterans of the war in Indochina in 1948. Afterwards, he expanded the use of lavender EO to treat agitation in psychiatric patients (Thomas, 2002). Recently, ethno‐pharmacological studies reported the medical usage of lavender EO to treat stress, anxiety and depression throughout the world (McIntyre et al., 2016; Xu et al., 2008).

However, the composition of EO still remains an issue to be disentangled, since each component could produce different psychoactive effects. To overcome this problem, the International Standard Organization (ISO) established specific standards for extraction and analytical composition in order to define EO quality grades (Baser et al., 2011; Lawrence, 1993; Maštovská & Lehotay, 2003), and in 2012, the European Medicines Agency (EMA) Committee on Herbal Medicinal Products (HMPC) adopted a community herbal monograph on *Lavandula angustifolia* Miller aetheroleum, providing specific posologies and oral administration methods. The specific HMPC assessment concluded that lavender oil showed a possible effect on several clinical conditions, asserting that its use in the medical field can be based on its long‐standing usage (EMA & HMPC, 2012). A promising way to exploit the beneficial effects of lavender is the inhalatory administration due to the fact that the olfactory pathway is a direct and rapid way to the brain (Erdő et al., 2018). The nasal route of drug administration has several advantages over oral or intravenous administration, which include non‐invasiveness, self‐administration, shorter time of effects onset and higher bioavailability due to avoidance of hepatic first‐pass metabolism. Moreover, bypassing the blood brain barrier may potentially increase central nervous system (CNS) availability of the drug (Dufes et al., 2003).

From a cognitive standpoint, the above‐mentioned processes might be involved in the frequently reported anxiolytic effects induced by lavender (Arslan et al., 2020; Kianpour et al., 2016; Yayla & Ozdemir, 2019), and, at the same time, this could be linked to the empowerment of cognitive functions, as some studies investigating the effects of anesthetic drugs on cognition reported an improvement in attention and memory domains (Mirski et al., 2010). Since according to the Yerkes–Dodson law (Yerkes & Dodson, 1908) cognitive performance is known to be optimal when arousal is at an intermediate level and, if that threshold is overtaken, performance decreases, low doses of lavender EO might induce arousal decrease to achieve more optimal levels in cognitive tasks, while higher doses of lavender EO might induce the well‐known anxiolytic effects.

Furthermore, a systematic and integrated study on the impact of lavender EO inhalation on cognitive functions is needed, since most studies focused on compartmentalized outcomes ranging from phytochemical composition of EO to physiological and behavioral measures. As regards lavender EO composition, linalool and linalyl acetate are the two main components whose mechanism of action is responsible for central (i.e., brain rhythms) and peripheral (i.e., autonomic tone) effects (Umezu et al., 2006; Zhong et al., 2019). Based on these premises, a systematic characterization of the effects of lavender EO on cognition from a multidisciplinary perspective would help in the comprehension of the cognitive enhancing effects of lavender EO inhalation, meaning a reduction in reaction times or an increase in task accuracy. In this context, different types of EO diffusion devices may also have an impact on administration procedure, since EO quality can be differentially affected by the employed device (Halligudi & Al Ojaili, 2013). Thus, taking into account the EO phytochemical parameters and administration methods, the aim of this systematic review is to assess in healthy subjects whether different lavender EO compositions can differentially influence core cold cognitive functions such as vigilance and arousal, attention and memory or whether these alleged effects hold true regardless of predetermined EO composition.

## MATERIALS AND METHODS

We carried out this systematic review according to the Preferred Reporting Items for Systematic Reviews and Meta‐Analyses (PRISMA) guidelines, which comprises a checklist to ensure the quality of systematic reviews (Moher et al., 2009), reported in Table S1. The protocol for the present systematic review has been submitted for registration (ID number 180709) to PROSPERO international prospective register for systematic reviews database (Moher et al., n.d.).

In order to develop an effective search strategy, we adopted the Population, Intervention, Comparison, Outcomes and Study design (PICOS) strategy (da Costa Santos et al., 2007), reported in Table S2. The literature search was conducted in August 2021 and included three phases: (1) identification: a systematic search based on queries on the electronic databases MEDLINE, ERIC, PsycInfo, Google Scholar, and Scopus of articles until August 2021 (an example of the publication trend of articles on aromatherapy retrieved until 2021 is provided in Figures S1 and S2); (2) screening: a manual screening of the articles yielded in phase (1) by evaluating title and abstract only; (3) final eligibility: a more in‐depth assessment of the full text of the remaining papers on the basis of the following eligibility criteria: (i) healthy humans of any age; (ii) inhalation as method of lavender EO administration because inhalation is the only non‐invasive method of administration through which the highest bioavailability of the substance is reached; (iii) behavioral and/or physiological assessment in order to exclude biases ascribable to self‐report measures; (iv) randomized‐controlled design; (v) articles published in peer‐reviewed journals; (vi) articles written in English language. At phase 1, boolean operators “AND” and “OR” were applied to combine a list of keywords related to lavender and a list of keywords related to cognitive functions retrieved from Cognitive Atlas website (Poldrack, 2011), reported in Table S3.

Table S4 summarizes the search steps performed on the database. Articles retrieved from MEDLINE, ERIC, PsycInfo, Google Scholar, and Scopus databases were merged into the Mendeley database (Mendeley Support Team, 2011), and duplicates were automatically removed using the desktop Mendeley reference manager. During the inclusion phase, we excluded articles containing words related to agricultural and botanical fields and to medical treatments in order to do a preliminary selection of the studies by focusing on procedures that employed EO in the field of cognitive functions research; to this aim, we used a list of excluding words related to the aforementioned fields to automatically exclude such studies (list of excluding words is reported in Table S5)

To assess the quality of evidence of the studies, we used the Newcastle‐Ottawa Scale (NOS, Table 2) for the evaluation of randomized‐controlled studies (Wells et al., 2000). The scale scores the quality of reporting with a maximum of 9 stars for higher quality. According to NOS, each study is judged for the selection of the study groups, comparability of the groups, and ascertainment of either the exposure or outcome of interest. In this process, two independent reviewers (E.M. and V.C.) scored each study according to NOS criteria. Any disagreement was resolved through consensus of all authors.

## RESULTS

**FIGURE 1:**
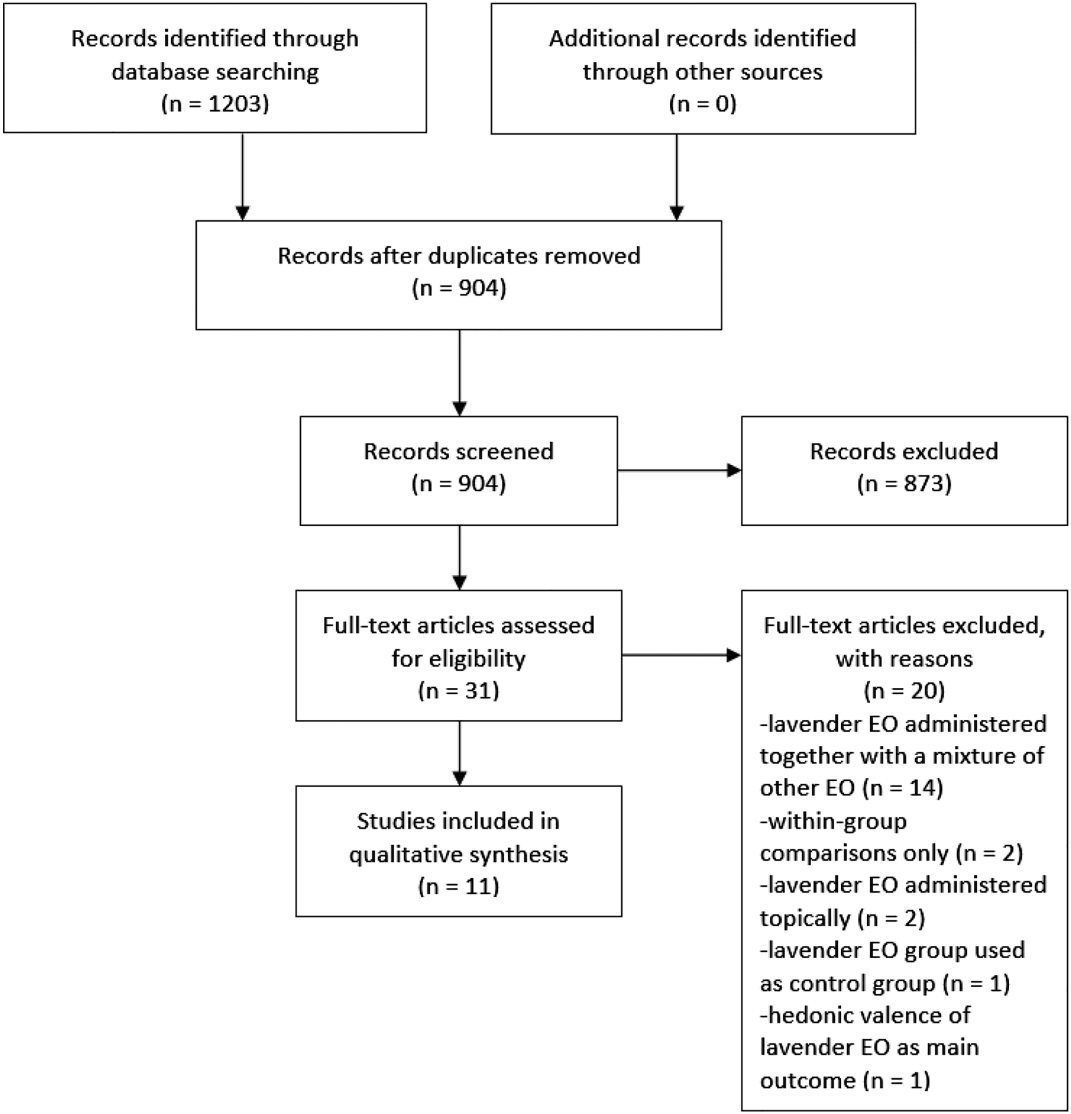
Flow chart of the selected studies

Our search yielded 1,203 articles. After duplicates removal and screening phase, 31 full‐text articles were retained according to the eligibility criteria. Twenty of them were excluded for the following reasons: 14 studies included mixed EO; one study did not investigate cognitive functions; one study used lavender EO inhalation as a control condition; 2 studies used topical application of lavender EO; 2 studies were based on a within‐group without randomized‐controlled design. The articles selection process is illustrated in Figure 1. After the inclusion phase, by evaluating the studies' outcomes, articles were divided into three macro‐categories regarding psychological macro‐domains: *arousal*, *attention*, and *memory*. Detailed information is provided in Table 1. Finally, 11 articles were reviewed. NOS scores were adequate for all studies and ranged from 5 to 7 (Table 2). According to Table 1, all studies recruited young adults and adults, except for two studies (Chamine & Oken, 2016; Heuberger & Ilmberger, 2010) in which elderly subjects were recruited too. None of the reviewed studies investigated either an age effect or effects elicited by different EO administration timing. Only two studies (Kim et al., 2011; Shimizu et al., 2008) investigated the temporal range in which cognitive effects are still detectable from inhalation onset.

**TABLE 1:** Cognitive and phytochemical primary outcomes

Study	Design	Subjects number	Age	Groups	Procedure	Measurement	Results for all groups	Odor awareness	EO characteristics	EO quali‐quantitative analyses	Diffusion system
**AROUSAL**											
Kiecolt‐Glaser et al., 2008	Single blind/not blind, crossover	18	Young adults, adults	Lavender, lemon, control (blind and primed subgroups for each group)	Physiological parameters were assessed before and after subjects underwent cold pressor test for one minute while inhaling EO	Norepinephrine	Decrease in lavender group compared to lemon group (*p* = 0.019)	Aware	PVE	GC–MS	c
						BP	No changes				
						HR	Increase in primed groups compared to blind groups (*p* = 0.002)				
						Salivary cortisol	Decrease in blind groups compared to not blind groups (*p* = 0.002)				
						Blastogenesis response: lymphocyte response to mitogens	Decrease in primed groups compared to water group (*p* = 0.001) and lemon group (*p* = 0.009)				
						Interleukin‐6 and ‐10	No changes				
						Delayed hypersensitivity to candida	Decrease in lavender group compared to water group (*p* = 0.02)				
Kim et al., 2011	Cross‐sectional, double blind, RCT	15	Young adults, adults	Lavender, control (passive)	Bispectral index was assessed before and at 5, 10, 15 and 20 min after 5 min of EO administration while subjects were resting	EEG bispectral index	Decrease at 5, 10, 15 and 20 min after inhalation, but not at 25 min in lavender group compared to control group (*p* < 0.001)	Aware	DVE	Not specified	cm
Saiorwan et al., 2012	Single blind, crossover	10	Young adults	Lavender, control (passive)	Physiological parameters were assessed at baseline and after 4 7‐min sessions of EO administration while subjects were resting	RR	No changes	Aware	DVE	GC–MS	cm
						HR	Decrease in lavender group compared to control group (*p* < 0.001)				
						SBP	Decrease in lavender group compared to control group (*p* < 0.001)				
						DBP	Decrease in lavender group compared to control group (*p* < 0.001)				
						Skin temperature	Decrease in lavender group compared to control group (*p* < 0.001)				
						EEG	Decrease in lavender group compared to control group (*p* < 0.05)				
**ATTENTION**											
Chamine et al., 2016	Single blind/not blind, RCT	31	Adults, elderly	Lavender, placebo (water or coconut), control (primed and not primed subgroups for each group)	Performance on a battery of cognitive tests was evaluated at baseline and after exposure to a 30‐min stress battery during which aromatherapy was administered	Stroop Color‐Word Test	No changes	Aware/unaware	DED	Not specified	c
						WAIS‐III: Simple reaction times	No odor effect, improvement in primed subgroups compared to not primed subgroups only (*p* < 0.05)				
Shimizu et al., 2008	Single blind, crossover	7	Young adults	Lavender, eucalyptus, control (passive)	Performance on a 30‐min vigilance task was assessed every 2 min during EO administration	Long‐term vigilance task	Attention increase at 14, 16, 26 and 30 min after EO administration onset in lavender group compared to control group (*p* < 0.001)	Aware	PVE/TCD	Not specified	cm
Sakamoto et al., 2005	Single blind, RCT	12	Young adults	Lavender, jasmine, control (passive)	Subjects performed 5 60‐min sessions of a sustained attention task and were administered with EO during 20‐min intervals between every session	Sustained attention task: reaction times	Attention increase in lavender group compared to control and jasmine groups (*p* < 0.05)	Aware	PVE	Not specified	g
						Sustained attention task: errors number	Attention increase in lavender group compared to control group (*p* < 0.007) and control group (*p* < 0.044)				
Heuberger & Ilmberger, 2010	Single blind, RCT	30	Young adults, adults, elderly	1,8 cineole, jasmine, peppermint, linalyl acetate 5 μl and 20 μl, control (passive)	Performance on a vigilance task was assessed during EO administration	Standard visual vigilance task: reaction times difference	Attention increase in 20‐μl linalyl acetate group compared to control group (*p* < 0.05)	Aware	PVE	Not specified	cm
						Standard visual vigilance task: scores of misses	No changes				
Moss et al., 2003	Single blind, RCT	48	Young adults	Lavender, rosemary, control (passive)	Performance on CDR battery was assessed during EO administration	CDR battery: speed of attention	Attention decrease in lavender group compared to control group (*p* < 0.05)	Unaware	PED	not specified	g
						CDR battery: accuracy of attention	No changes				
Colzato et al., 2014	Single blind, RCT	22	Young adults	Lavender, peppermint, control (passive)	Attentional blink in a 10‐min RSVP task was assessed during EO administration	RSVP task	Less pronounced attentional blink in lavender group compared to peppermint group (*p* = 0.043)	Unaware	PED/DED	Not specified	g
Sellaro et al., 2015	Single blind, RCT	24	Young adults	Lavender, peppermint, control (passive)	Performance at Joint Simon task was evaluated during EO administration	Joint Simon task (correspondence trials): errors	No changes	Unaware	PED	Not specified	g
						Joint Simon task (correspondence trials): reaction times	No changes				
						Joint Simon task (noncorrespondence trials): errors	Attention decrease in lavender group compared to control group (*p* < 0.05) and peppermint group (*p* < 0.005)				
						Joint Simon task (noncorrespondence trials): reaction times	No changes				
						Joint Simon task: Simon effect (errors)	Attention decrease in lavender group compared to control and peppermint groups (*p* < 0.05)				
Yue et al., 2016	Single blind, RCT	23	Young adults	Lavender, jasmine, garlic, control (passive)	Performance on a perception of time task was assessed during EO administration	Time reproduction task	Attention increase in lavender group compared to jasmine group (*p* < 0.01) and garlic group (*p* < 0.05)	Unaware	Not applicable	Not specified	g
**MEMORY**											
Chamine et al., 2016	Single blind/not blind, RCT	31	Adults, elderly	Lavender, placebo (water or coconut), control (primed and not primed subgroups for each group)	Performance on a battery of cognitive tests was evaluated at baseline and after exposure to a 30‐min stress battery during which aromatherapy was administered	WAIS‐III: digit span backward	Memory improvement in lavender group compared to coconut group (*p* = 0.008) and water group (*p* = 0.021)	Aware/unaware	DED	Not specified	c
Moss et al., 2003	Single blind, RCT	48	Young adults	Lavender, rosemary, control (passive)	Performance on CDR battery was assessed during EO administration	CDR battery	Memory decline in lavender group compared to control group (*p* < 0.01) and rosemary group (*p* < 0.05)	Unaware	PED	Not specified	g

Abbreviations: EO, essential oil; BP, blood pressure; EEG, electroencephalogram; WAIS‐III, Wechsler Adult Intelligence Scale‐III; PVE, pure volatile essential oil; CDR, cognitive drug research; RSVP, rapid serial visual presentation; PED, pure essential oil drops; RCT, randomized‐controlled trial; HR, heart rate; RR, respiratory rate; SBP, systolic blood pressure; DBP, diastolic blood pressure; DVE, diluted volatile essential oil; DED, diluted essential oil drops; TCD, target compound diffusion; GC‐MS, gas‐chromatography mass‐spectrometry; c, cotton pad/gauze/linen fabric (two layers under nose, direct diffusion system); cm, cotton pad/gauze/linen fabric/ball in surgical mask and oxygen flow/inhalers (direct diffusion system); g, candle diffuser/soaked pad under bench/tank‐becker (no electrical diffuser, indirect diffusion system).

**TABLE 2:** Newcastle Ottawa Scale for quality assessment of the included studies

Author and Year	Selection				Comparability	Exposure			Total score quality
	Case definition	Representativeness of the cases	Selection of controls	Definition of controls	Comparability of cases and control	Ascertainment of exposure	Same method of ascertainment for cases and controls	Non‐Response rate	
Kiecolt‐Glaser et al., 2008	**⋆**		**⋆**	**⋆**	**⋆⋆**	**⋆**	**⋆**		**7**
Kim et al., 2011			**⋆**	**⋆**	**⋆**	**⋆**	**⋆**		**5**
Saiorwan et al., 2012	**⋆**		**⋆**		**⋆**	**⋆**	**⋆**		**5**
Chamine et al., 2016		**⋆**	**⋆**	**⋆**	**⋆⋆**	**⋆**	**⋆**		**7**
Colzato et al., 2014		**⋆**	**⋆**	**⋆**	**⋆**	**⋆**	**⋆**		**6**
Heuberger & Ilmberger, 2010		**⋆**	**⋆**	**⋆**	**⋆**	**⋆**	**⋆**		**6**
Moss et al., 2003		**⋆**	**⋆**	**⋆**	**⋆**	**⋆**	**⋆**		**6**
Sakamoto et al., 2005			**⋆**	**⋆**	**⋆**	**⋆**	**⋆**		**5**
Sellaro et al., 2015		**⋆**	**⋆**	**⋆**	**⋆**	**⋆**	**⋆**		**6**
Shimizu et al., 2008			**⋆**	**⋆**	**⋆**	**⋆**	**⋆**		**5**
Yue et al., 2016		**⋆**	**⋆**	**⋆**	**⋆**	**⋆**	**⋆**		**6**

### Phytochemical and diffusion parameters

**FIGURE 2:**
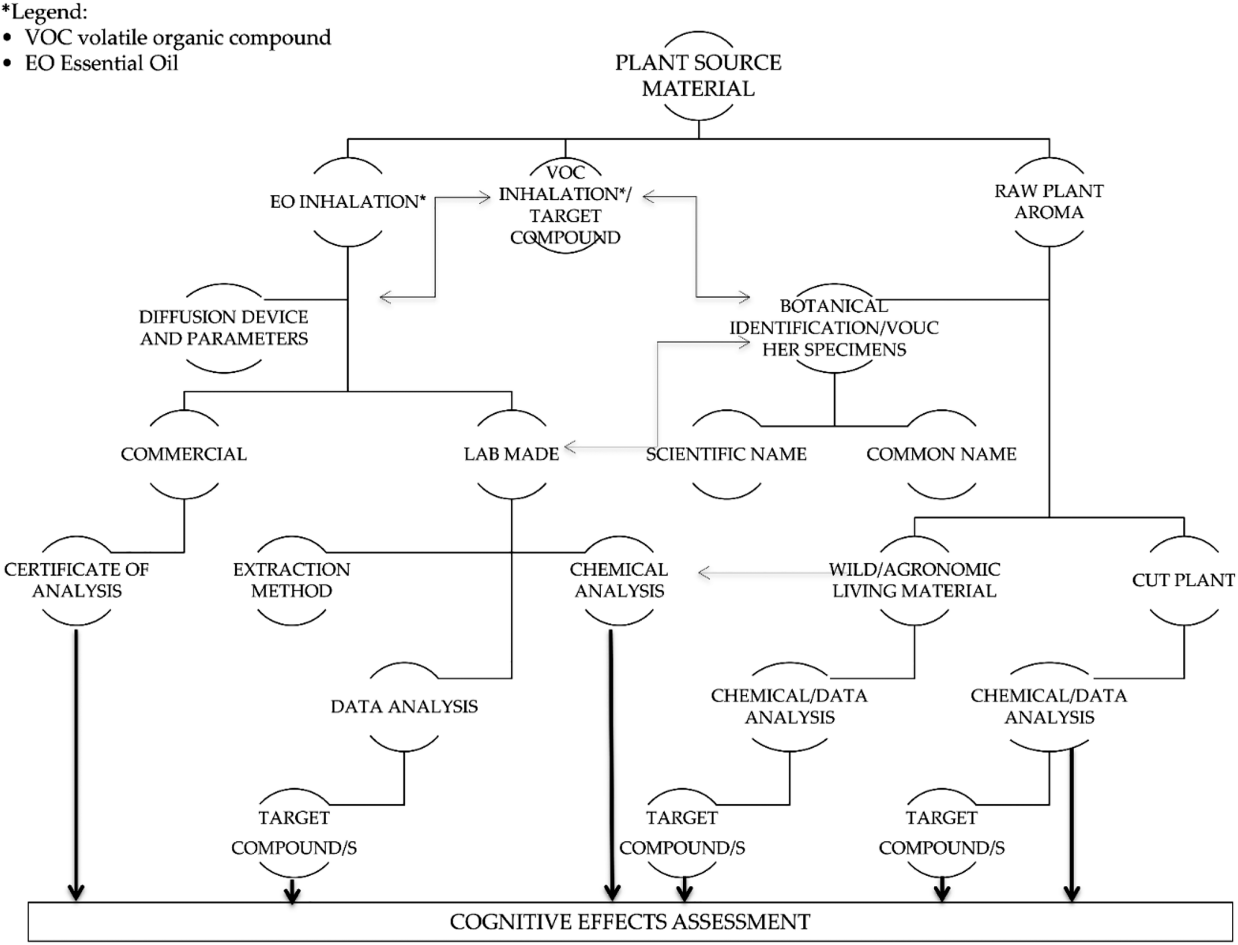
Flow diagram illustrating the quality assessment of the inhaled sample: EO composition and administration systems evaluation as a preliminary step leading to cognitive effects assessment

In the revision process, the selected articles were reviewed by taking into account four key points of EO selection and aroma diffusion: (i) quality parameters reported either in commercial documentation or lab‐scale distilled EO (i.e., pure EO samples obtained by distillation using lab equipment such as Clevenger apparatus recommended by European Directorate, 2019), (ii) sample preparation procedures, (iii) diffusion devices and (iv) subjects' awareness towards administration procedure depending on the device used. Selection process is illustrated in Figure 2.

Quality parameters and sample preparation procedures were not always reported in the selected studies (Colzato et al., 2014; Moss et al., 2003; Sellaro et al., 2015). Different aromatic products were used by Heuberger and Ilmberger (2010) such as commercial singular volatile compounds, EO and absolute ether extract, which are referred to by the general term “fragrances”. The authors did not specify either if the EO were synthetic or isolated from plant material or the quality analytical results of EO purity and composition. Although Kiecolt‐Glaser et al. (2008) did not report compositional data, theirs was the only experimental procedure based on lavender EO monitoring in order to avoid compositional variability. In Saiorwan and colleagues' study (2012), the quality control of commercial lavender EO (i.e., pure or diluted EO or mixtures of volatile chemicals sold on the current international market) was carried out by gas chromatography–mass spectrometry analysis, which is an analytical technique used both in industrial and research laboratories to analyse EO samples. It combines chromatographic separation on a selected column with mass spectrometry detection in order to define the EO chemical profile by both a fixed retention time for each volatile on that selected column and a specific mass spectrum. Identification process is performed by the comparison of the obtained experimental retention time/mass spectrum with those of pure compounds. However, only the relative percentages of linalool (31.9%) and linalyl acetate (32.5%) were shown. Despite these two biomarkers (i.e., the typical volatile components of lavender EO linked to a specific bio‐pharmacological action) being detected in the same amount, the authors considered only linalool as the main compound whose effects on cognitive functions were studied. In other cases, the scientific botanical name of the lavender raw material was the only certified information and the EO composition was not reported at all (Chamine & Oken, 2016; Sellaro et al., 2015). Shimizu et al. (2008) reported no information about linalyl acetate level in the tested EO sample, although the study considered the singular EO compound linalyl acetate as the main active lavender constituent.

Regarding diffusion devices, a variety of facilities with different diffusion methods have been described in the studies. According to EO administration method, two types of devices were identified: (1) direct administration (face systems) including steam diffusion, mask, natural volatile system lab‐scale and cotton pad under nose; (2) indirect administration (environmental systems) including cotton pad under seat, candle and electric devices. Direct administration methods allowed subjects to be aware of the procedure, while indirect administration methods prevented subjects from being aware of the protocol they had been involved in (see Table 1).

Six studies employed direct EO administration systems (Chamine & Oken, 2016; Heuberger & Ilmberger, 2010; Kiecolt‐Glaser et al., 2008; Kim et al., 2011; Saiorwan et al., 2012; Shimizu et al., 2008). Shimizu et al. (2008) used a lab‐scale Natural Volatile Delivery System to spread lavender EO and linalyl acetate in the room environment, avoiding dishomogeneous EO dispersion. This device was placed 10 centimeters below the nose of each subject and allowed EO flow at constant pressure and quantity (amount per liter of air). Heuberger and Ilmberger (2010), Kim et al. (2011) and Saiorwan et al. (2012) administered EO by means of inhalation from a face mask. In particular, Heuberger and Ilmberger (2010) applied to subjects a surgical mask coated with lavender EO; Saiorwan et al. (2012) mixed volatile components of EO with oxygen through a pump connected to an oxygen mask; Kim et al. (2011) applied a cotton swab soaked with EO to an oxygen mask. No indication about the operating oxygen mask flow has been described. Kiecolt‐Glaser et al. (2008) and Chamine and Oken (2016) used tissue diffusion method by applying a cotton swab below the subjects' nose. In their study, Kiecolt‐Glaser et al. (2008) used a commercial lavender EO, identified by its specific tradename and producer, diluted in water (four drops**/**30 ml).

Five studies used indirect EO administration systems (Colzato et al., 2014; Moss et al., 2003; Sakamoto et al., 2005; Sellaro et al., 2015; Yue et al., 2016). Sakamoto et al. (2005) and Yue et al. (2016) used general evaporative methods to diffuse lavender and jasmine EO samples. Authors also described the adequate room parameters to guarantee insulation conditions and homogenous EO concentration in the room. A testing aromatic delimited room was also used by Moss et al. (2003), who employed a dedicated cubicle for a more controlled scent room by applying a soaked cotton pad in a container under the subject's seat. Colzato et al. (2014) and Sellaro et al. (2015) administered EO by means of a candle for a fixed exposure time.

### Effects of lavender EO inhalation on arousal

Three studies investigated lavender aromatherapy effects on arousal in healthy subjects (Kiecolt‐Glaser et al., 2008; Kim et al., 2011; Saiorwan et al., 2012). Physiological measurements were assessed at baseline and after exposure to EO. In the studies by Kim et al. (2011) and by Saiorwan et al. (2012), participants inhaled EO while resting, whereas, in Kiecolt‐Glaser and colleagues' study (2008), participants underwent a stress‐response inducing test.

Kiecolt‐Glaser et al. (2008) found complex results while studying physiological responses to the cold pressor test as a function of inhaled EO while manipulating odor expectancy (priming). In their experiment, they found that norepinephrine levels following the cold pressor test diminished in water and lavender conditions compared to lemon condition; delayed hypersensitivity to candida, an index of T‐cells immunity, and blastogenesis response to mitogens, that are markers of stress‐arousing response, were smaller after lavender condition compared to water condition. Primed and blind groups showed no differences. Blastogenesis response to mitogens was higher in lavender primed group than in water and lemon primed groups. EO did not alter the following stress‐related parameters: interleukin‐6 and interleukin‐10 levels, salivary cortisol, heart rate (HR) or blood pressure (BP); however, expectancy effect was revealed for HR, since primed groups showed higher HR increase after cold pressor test, but also larger HR decrease during recovery as compared to blind groups.

Saiorwan et al. (2012) assessed central and peripheral correlates of arousal level. EEG measures revealed fronto‐central and temporal increase of power in alpha and theta bands, which, as the authors state, are indices of arousal decrease,while autonomic parameters showed reduction of HR, systolic and diastolic BP and skin temperature in the lavender group compared to the control group (administered with base oil), but no differences were shown in respiratory rate.

Kim et al. (2011) assessed arousal with the bispectral index (BIS), an electroencephalogram derivative index which reflects the level of consciousness during sedation and acupuncture (Fassoulaki et al., 2003; Liu et al., 1996) while inserting a needle into the forearm skin to induce a stress‐arousing state in healthy participants. Authors found a decrease of arousal in the lavender EO group compared to the control group, whose participants were administered with oxygen only. Between‐group differences were significant at 5, 10, 15, and 20 but not at 25 min after the end of EO inhalation.

### Effects of lavender EO inhalation on attention

Eight studies investigated the effects of lavender EO on attention in healthy subjects (Chamine & Oken, 2016; Colzato et al., 2014; Heuberger & Ilmberger, 2010; Moss et al., 2003; Sakamoto et al., 2005; Sellaro et al., 2015; Shimizu et al., 2008; Yue et al., 2016). All studies assessed performance on cognitive tasks while subjects were inhaling EO, except for Sakamoto and colleagues' study (2005), in which participants were administered with EO during intervals between cognitive task sessions.

Chamine and Oken (2016) implemented a mixed design in which lavender and coconut groups were divided into two subgroups: one group was told it would receive a relaxing aroma, the other was told that the aroma might have relaxing effects or not. Control group was administered with water and received no priming. Participants then underwent a stress battery including emotional and physical stressors while inhaling lavender or coconut EO or distilled water. Authors conducted cognitive and physiological assessment to detect the effects of EO controlling for expectation. Authors did not find significant changes after EO inhalation for Stroop Color‐Word test (Stroop, 1935) (a task in which the color of the ink of a list of color names does not match with the semantic significance of the words and subjects are required to say the name of the ink, instead of reading the color name) and WAIS‐III Letter‐Number Sequencing (Wechsler, 1997) (a task in which participants must first say the numbers in ascending order and then the letters in alphabetical order), but faster reaction times were detected among primed subgroups, regardless of the EO. Physiological assessment showed changes in cortisol level and in respiratory rate, but chromogranin A level, a reliable marker of psychological stress, was found to be lower in the control group as compared to both EO groups.

Shimizu et al. (2008) reported faster reaction times during a sustained attention task after lavender EO administration, compared to the control group that received no odor. Between‐group differences were significant at 14, 16, 26, and 30 min after EO administration onset.

In Sakamoto et al.'s (2005) study, a sustained attention task was performed by participants, showing that, compared to jasmine and no‐odor groups, lavender EO produced faster reaction times and improved accuracy during all task sessions.

The study by Heuberger and Ilmberger (2010) showed faster reaction times during a vigilance task in participants who had inhaled 20 μl of linalyl acetate as compared to those in the control group who were administered with water. This result was not significant for 5 μl of linalyl acetate. Reaction times decrease correlated with subjective ratings of fragrance: the more pleasant the lavender fragrance was rated, the faster reaction times were. Few errors correlated with subjective ratings too: the more stimulating the water, the fewer the errors.

On the contrary, Moss et al. (2003) reported slower reaction times induced by lavender EO inhalation during a vigilance task, compared to rosemary and no‐odor groups.

Colzato et al. (2014) investigated the effects of lavender and peppermint EO inhalation on the attentional blink, that reflects temporal limitations in visual attention allocation, assessed with RSVP task (Potter, 1975), a task in which subjects are required to detect a target stimulus embedded in a stream of visual stimuli. Attentional blink was found to be less pronounced among participants in the lavender group as compared to the control group, which did not undergo any intervention. The authors of the study interpreted the result as a less focused attentional state induced by lavender.

Sellaro et al. (2015) investigated Simon effect (Simon & Rudell, 1967) in the following three groups: participants inhaling lavender EO; participants inhaling peppermint EO; participants exposed to no aroma. This effect is the difference in accuracy or reaction times between trials in which stimulus and response are on the same side and trials in which they are on opposite sides, with responses being generally slower and less accurate when stimulus and response are on opposite sides; namely, the subject is required to manage spatially‐conflicting stimuli to provide the correct motor response: indeed, the task assesses the ability to suppress automatic response when the localization of the stimulus is different from the localization in which the response is required. More errors in the stimulus/response locations discordance condition were found in the lavender group, compared to peppermint or control groups.

Yue et al. (2016) investigated how time perception varied depending on different EO inhalation, as a measure of odor‐dependent attentional deployment. The authors found that, during the time tracking task, subjects perceived longer time duration than the actual time duration in the lavender group, compared to garlic and jasmine groups.

### Effects of lavender EO inhalation on memory

Two studies investigated lavender EO effects on memory in healthy subjects, reporting different results (Chamine & Oken, 2016; Moss et al., 2003). Performance on cognitive tasks was assessed during EO administration.

Chamine and Oken (2016) found working memory performance improvement after lavender inhalation as compared to placebo and water inhalation; memory was assessed by WAIS‐III digit span backward (Wechsler, 1997). Neither priming nor placebo effects were reported. However, Moss et al. (2003) showed that participants who had inhaled lavender EO had worse working memory performance as compared to the rosemary group and to the control group that received no odor. Working memory was assessed by CDR battery (Wesnes et al., 1999), which comprises numerical working memory and spatial working memory tasks; for both tasks, speed and accuracy were measured.

## DISCUSSION

**FIGURE 3:**
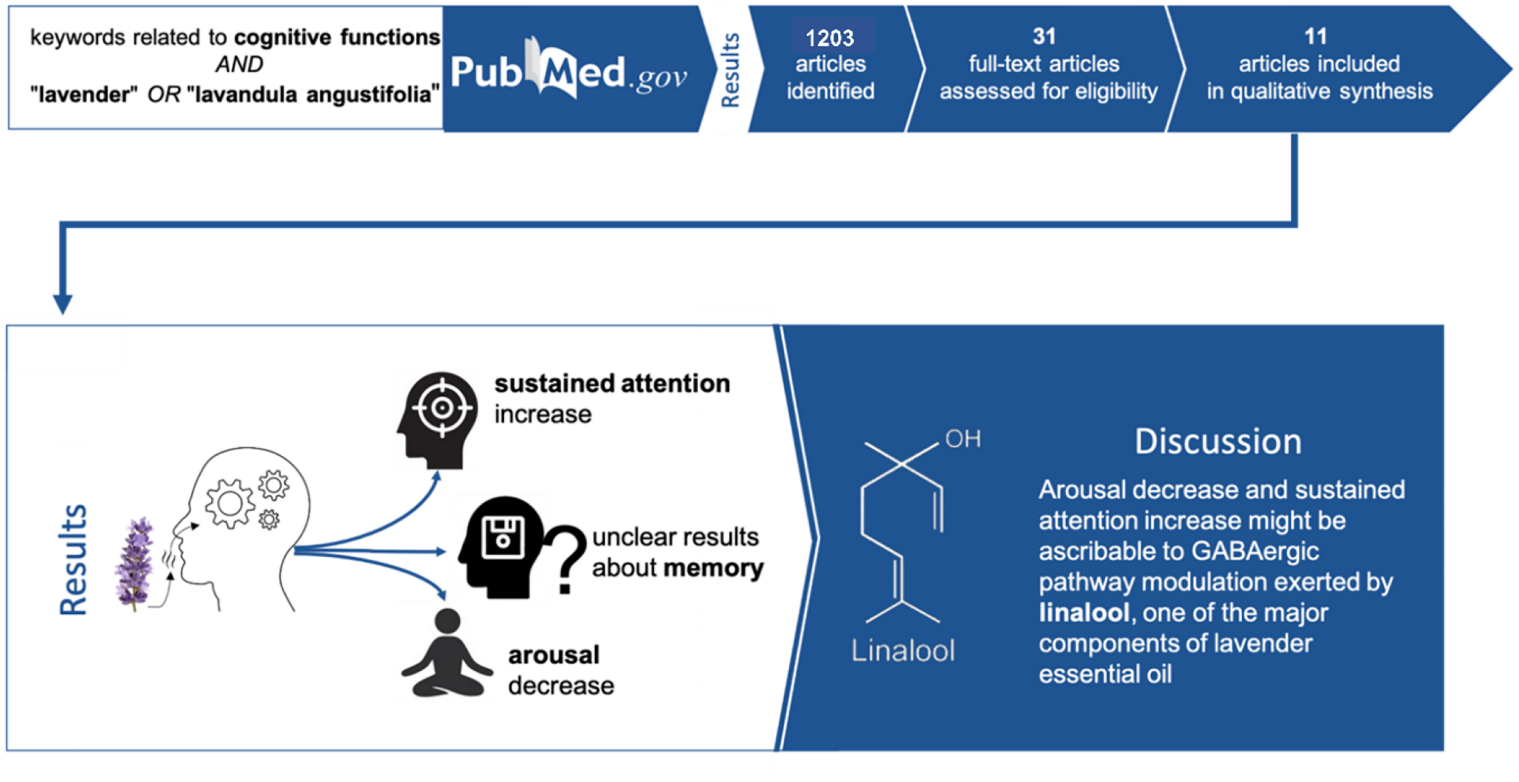
Graphical representation of the systematic review process [Color figure can be viewed at wileyonlinelibrary.com]

This systematic review aims to provide a comprehensive qualitative synthesis of psychophysiological outcomes of lavender EO inhalation, taking into consideration its quality issues as well as diffusion systems (Figure 3).

### Lavender EO and cognition

As regards the effects of lavender EO inhalation on attention, the most frequent result emerging from the studies was reaction times decrease during sustained attention tasks (Sakamoto et al., 2005; Shimizu et al., 2008; Yue et al., 2016). These findings, together with less pronounced attentional blink found by Colzato et al. (2014), suggest that lavender might induce a temporally distributed attentional state, which positively influences performance in sustained attention tasks, at the expense of focused attentional state, as shown in Sellaro and colleagues' study (2015): in fact, lavender produced a more pronounced Simon effect, which is elicited by a task implying conflict monitoring abilities (Simon & Rudell, 1967).

However, Chamine and Oken (2016) showed that faster reaction times in a vigilance task were induced by the expectancy effect, since high expectations led to cognition enhancement, regardless of intrinsic properties of lavender. These results are coherent with Heuberger and Ilmberger's (2010) study, which found that increase of performance speed correlated with subjective ratings of odor. Therefore, these studies suggest that priming and judgement of the odor value were responsible for cognition enhancement instead of lavender EO itself.

The aforementioned findings might be related to the arousal outcomes. The experimental procedures of these studies implied a stress‐inducing situation leading to arousal increase, which led to performance detriment and which was found to be reversed by lavender inhalation. Indeed, Saiorwan et al. (2012) found a decrease in autonomic parameters and an increase of power in alpha, beta and theta bands during lavender EO inhalation, indicating arousal decrease. These findings are corroborated by one EEG study which reported increased alpha waves after lavender EO inhalation, suggesting a relaxing effect (Park et al., 2019). Moreover, three studies (Diego et al., 1998; Dimpfel et al., 2004; Songsamoe et al., 2020) showed a positive correlation between lavender EO inhalation and beta power increase, indicating arousal decrease; in the same study, arousal decline allowed the subjects to better perform sustained attention tasks. It is worth noting that Diego et al. (1998), Dimpfel et al. (2004), Park et al. (2019) and Songsamoe et al. (2020) also studied cognitive effects of lavender EO; however, these studies were not included in this systematic review because they were based on a within‐subject design. Moreover, beta power is reported to be also related to less pronounced attentional blink (Gross et al., 2004; Shapiro et al., 2017), and this supports the hypothesis of a possible causal relation between lavender EO inhalation, arousal decrease and higher tonic attention performance. Indeed, as the Yerkes and Dodson (1908) law states, arousal decrease induced by lavender EO inhalation might have produced performance empowerment in sustained attention tasks.

As regards the few and controversial results about working memory, its improvement reported by Chamine and Oken (2016) might be explained by Yerkes and Dodson law (Yerkes & Dodson, 1908), as well. Indeed, arousal decline induced by lavender can be considered as a cognitive substrate which promotes the deployment of higher cognitive functions, including working memory, whose structural and functional correlates partly overlap with those of attention (deBettencourt et al., 2019; Petersen & Posner, 2012). However, Moss et al. (2003) did not confirm the previous findings, reporting working memory decline after lavender EO inhalation. This incoherence might reflect the use of different neuropsychological tools to assess working memory: Chamine and Oken (2016) employed WAIS‐III backward digit span (Wechsler, 1997), while Moss et al. (2003) used CDR battery (Wesnes et al., 1999). It is well stated that a certain degree of task impurity can affect neuropsychological tasks. In this context, working memory subscale of CDR battery might be more specific for working memory assessment than WAIS‐III backward digit span.

The previously‐mentioned results suggest potential central neural correlates of odor‐induced effects. Focusing on lavender EO mechanism of action, the principal component of lavender is the acyclic monoterpene linalool, which has been shown to have GABA A receptors as main targets (Hossain et al., 2002; Milanos et al., 2017). In fact, volatile components are reported to bind to odorant receptors at the olfactory epithelium (Buck, 1993). Harada et al. (2018) confirmed that linalool effect was mediated by GABA, since it was antagonized by flumazenil. Since the GABAergic circuit has been found to actively modulate arousal in locus coeruleus (Breton‐Provencher & Sur, 2019), it is possible to assume that lavender EO might induce arousal decrease acting through the GABAergic pathway. Moreover, this effect was not observed in anosmic mice, indicating that it was triggered by olfactory input evoked by linalool (Harada et al., 2018).

We can speculate that arousal decrement might be related to top‐down processes: since orbitofrontal cortex receives projections from piriform cortex (Plailly et al., 2008; Reiner et al., 2020) and, together with anterior cingulate cortex, represents the only cortical structure to send efferent projections to locus coeruleus (Aston‐Jones & Cohen, 2005), arousal decrease might be induced by the modulation of locus coeruleus GABAergic neurons exerted by orbitofrontal cortex. In fact, neocortex is thought to be the main target of the sedative effects of volatile anesthetics, since its neurons are more sensitive to these chemical compounds (Becker et al., 2012; Hentschke et al., 2005). However, other possible neurobiological mechanisms of action of lavender volatile components are not to be excluded: since some evidence reported linalool to also act through the serotonergic pathway (Chioca et al., 2013; Lee et al., 2018), its effect could be ascribable to raphe nucleus activation by olfactory input from olfactory bulb (Miro et al., 2001), and subsequent bottom‐up process to cortical areas (Arguinchona & Tadi, 2021; Kandel, 1985).

However, since only one study (Chamine & Oken, 2016) included placebo condition, a placebo effect or an overlap between lavender EO mechanism of action and placebo effect are not to be definitely ruled out. Indeed, Masaoka et al. (2013) reported a decrease in pain perception induced by expectancy of the analgesic effects of lavender; as compared to the control group and subjects inhaling aroma without knowing it, this effect was more prominent in subjects who were informed they were inhaling an analgesic aroma and actually inhaled lavender EO, but it was also present among subjects who believed they were inhaling an analgesic EO but received no treatment. This study was not included in the systematic review because it investigated a psychological domain that does not concern the present study. Similarly, since placebo effect derives from subjective interpretation of context information and most of medical treatments benefits are caused by the brain's response to the treatment context (Wager & Atlas, 2015), visible EO diffusion devices might act as contextual cues, inducing an expectancy effect in subjects. However, the mere effect of aromatherapy is supported by some studies that highlighted cognitive enhancing effects of lavender EO in several clinical conditions that imply cognitive impairment, such as dementia and Alzheimer's disease (Ayaz et al., 2017).

Anatomo‐functional evidence corroborates the role of lavender EO inhalation in modulating cognitive functions through specific neuroanatomical pathways triggered by olfactory bulb. A recent study reported anatomical and functional connectivity between olfactory system and subcortical hubs, suggesting an indirect role of olfactory stimulation in arousal, reward learning, emotions and memory (Zelano et al., 2016); in particular, the stimulation of olfactory epithelium mechanoreceptors was found to lead to electrical activity synchronization of olfactory bulb, piriform cortex, hippocampus, and amygdala as well (Heck et al., 2019; Piarulli et al., 2018). Furthermore, the high degree of functional connectivity between olfactory systems and neocortex suggests that olfactory stimulation may play an important role in the modulation of higher cognitive functions, such as executive functions and attention (Cohen et al., 2015; Smith & Bhatnagar, 2019).

### EO quality control and intervention protocols

Due to the EO compositional complexity, chemical profile assessment should be considered as a preliminary crucial step to ensure quality and safety of research in aromatherapy.

Despite the importance of providing the gas chromatography–mass spectrometry composition, batch number and trade name of EO sample, in most of the reviewed papers, understanding the EO quality of the inhaled sample was not possible. In a few cases (Heuberger & Ilmberger, 2010; Saiorwan et al., 2012; Shimizu et al., 2008), only two main major constituents, linalool and linalyl acetate, have been described. However, the presence of other typical minor compounds has not been provided. In general, monitoring the EO compositional data is crucial in order to manage adverse effects, such as hepatotoxic effects exerted by some volatile constituents, namely, pulegone, menthofuran, camphor, limonene, zederone, and germacrone. These well‐known natural compounds are frequently used in traditional medicine, pharmaceutical industry and cosmetics. Furthermore, they exhibit high bioavailability through several ways of administration, including inhalation (Zárybnický et al., 2018). For this reason, in the case of lavender EO, chemical composition should be specifically reported even when the singular chemical compounds linalool or linalyl acetate are used, to guarantee purity and to exclude the presence of hazard or allergenic additives (ISO TC). Regarding the current official documentation, the community herbal monograph on *L. angustifolia* Miller aetheroleum, adopted by EMA, provided specific posologies, including oral administration methods, based on its long‐standing usage (EMA & HMPC, 2012). In addition, a further assessment report has also been recently required on *L. angustifolia* Mill. by the EMA to update research outcomes on safety of lavender in relieving stress and anxiety. No relevant further changes to the monograph were deemed necessary in consideration of lavender confirmed safety (EMA & HMPC, 2020). The World Health Organization restated that oral use of lavender is safe (WHO monographs on selected medicinal plants, 2007, 2018). Lavender scent has been tested in many research studies without any reported side effects (Kritsidima et al., 2010; Muzzarelli et al., 2006) and adverse skin reactions (Khan & Abourashed, 2011) have been observed with topical use only.

Lavender medical properties are especially due to its EO and in particular to its major volatile constituents linalool and linalyl acetate, which have recently been reported to prevent damages to liver (hepatoprotective properties) and kidneys (nephroprotective properties) (Altınok‐Yipel et al., 2020; Mazani et al., 2020; Mohamed et al., 2020). In addition, they showed no mutagenic activity in different *Salmonella typhimurium* strains with or without metabolic activation (Evandri et al., 2005).

Considering lavender EO composition complexity and variability, quali‐quantitative characterization is necessary to determine the contribution of each component. Focusing on the main lavender constituents, the ratio between linalool and linalyl acetate in the EO batch sample should be specified, as linalyl acetate derives from linalool during the extraction process. Furthermore, the monoterpene linalool (3,7‐dimethylocta‐1,6‐dien‐3‐ol) has generally been reported to influence the transmission of odor stimulation. In fact, the oxygen atom's structure allows hydrogen bond formation with a hydrogen atom on the olfactory receptor. In addition, the chemical relationship between linalyl acetate and linalool is really tight, because linalool is also the common and direct product of linalyl acetate hydrolysis together with acetic acid. Therefore, lavender EO activity could be strongly influenced by the linalyl acetate hydrolysis in favor of linalool levels followed by the unpleasant pungent odor of acetic acid (Akira, 2007).

Among the reviewed papers, Kiecolt‐Glaser et al. (2008) used lavender EO extracted by steam distillation in lab‐scale and analysed it before the intervention by gas chromatography–mass spectrometry. They monitored lavender EO also during the successive experimental sessions in order to avoid any compositional variability, but without reporting compositional data. Colzato et al. (2014) considered linalool as the main component of lavender EO and, most importantly, they suggested its role in attention increase: in this context, the authors only measured linalyl acetate as reference control. Both linalool and linalyl acetate have been reported to have complementary mechanisms which seem to be responsible for lavender EO cognitive effects (Cavanagh & Wilkinson, 2002; Degel & Koster, 1999). On the other hand, other studies reported only linalool to be responsible for nervous system modulation. Regarding the qualification of linalyl acetate as character impact compound reported in Heuberger and Ilmberger (2010), *L. angustifolia* Miller EO shows quite similar amounts of both linalyl acetate and linalool (ranges 7–56%, 6–50% respectively; generally 30–35%), following Pharmacopoeia (Pharmacopée Française X Édition, 1983, 1990) or ISO/AFNOR guidelines (ISO TC). Therefore, both these compounds have been believed to be active substances. Nevertheless, Heuberger and Ilmberger (2010) asserted linalyl acetate to be the main psychotropic active principle. In light of the linalool and linalyl acetate structure–activity relationship, the EO profile in toto has to be considered, due to the fact that minor constituents can also play a crucial role in olfactory stimulation, too. In addition, the authors used EO and an absolute ether extract introducing all of them by the general terms “fragrances”. They specified the related provider, but no mention was made of their being either synthetic or isolated from plant material, or of the quality control results of these materials in terms of EO composition.

Another important drawback is the wide variety of vehicles used to dilute the inhaled sample, since they could interfere with the lavender EO olfactory stimulation. In this context, Diego et al. (1998) diluted and administered lavender EO using grapeseed oil (10%), while Saiorwan et al. (2012) diluted lavender EO in sweet almond oil (10%) and Kim et al. (2011) used jojoba oil (2%) as vehicle.

To ensure homogeneous experimental procedures and treatment, the established and controlled exposition time should be associated with dimensioned room, air ventilation parameters and administration device, in order to allow protocol reproducibility and results comparability.

As regards diffusion systems, indirect administration represents a practical and cheap method that guarantees blindness towards procedure; however, while the oils volatile components are gradually released into the air, aroma quick dissipation may cause chemical degradation of some natural lavender constituents (Halligudi & Al Ojaili, 2013). For example, circumscribed steam diffusion (a tank with boiled water), used by Sakamoto et al. (2005), allows a quick release of aroma into the room, but it does not guarantee a long‐lasting and constant permanence of aroma in the air, since the use of heat may invalidate EO quality by disrupting its constituents (Halligudi & Al Ojaili, 2013). Furthermore, in order to guarantee a sufficient amount of aroma for a proper temporal range into the room, the tank used in the study was not small enough to ensure subjects' blindness towards procedure. Therefore, in this case, indirect administration method did not guarantee subjects' unawareness towards protocol. Concerning direct administration systems, cotton tissue generally supports the diffusion in other highly practical methods. Despite preventing EO from decomposition, cotton does not promote aroma diffusion into the air (Halligudi & Al Ojaili, 2013). This procedure has the advantage of avoiding volatile components dispersion, but it does not guarantee blindness towards procedure; moreover, a preliminary sensitivity test is recommended to avoid skin reactions. In fact, Kiecolt‐Glaser et al. (2008) considered their experimental system (soaked cotton ball with a fixed volume of EO tapped between nose and upper‐lip) better than environmental room inhalation, as it guarantees more continuous and homogeneous exposure. Heuberger and Ilmberger (2010), Kim et al. (2011) and Saiorwan et al. (2012) administered EO using a face mask. In particular, Heuberger and Ilmberger (2010) applied a surgical mask coated with lavender EO to subjects; Saiorwan et al. (2012) mixed volatile components of EO with oxygen through a pump connected to an oxygen mask; Kim et al. (2011) applied a cotton swab soaked with EO to an oxygen mask. These methods allow the maintenance of EO quality, preventing the environmental dispersion and possible contamination with other indoor volatiles. However, these advantages did not lead to subjects' blindness with respect to aroma diffusion. Therefore, direct and indirect EO diffusion systems can be considered as two complementary administration methods: on the one hand, if direct administration systems prevent EO from dispersion but may manipulate subjects' expectancy towards the procedure, on the other hand, indirect administration systems guarantee subjects' blindness but preclude EO components integrity.

### Limitations and Future Perspectives

Among the reviewed studies, several issues arise from a wide and unclear variety of terms used for the description of the administered EO. These issues are frequently observed in the field of complementary and alternative medicine, since several systematic reviews highlighted methodological flaws and heterogeneity of protocols (Kantor, 2009; Ng et al., 2018). This fact has an important impact on the evaluation of the effectiveness of the inhalation protocol. The selection of lab‐scale distilled EO, commercial lavender EO and marker composition should be based on the relative certificate of analysis in order to provide information linked to supplier and transformation process (plat raw material, extraction, carrier type, dilution ratio, process, batch number). Due to the frequent market adulteration for many plant derivatives, it is not possible to assume that every sold EO has been checked and passed quality requirements. In fact, botanical authenticity and certification results are crucial to guarantee the subject's safety to better understand the relationship between EO volatile components and odor‐induced effects on cognitive functions. In addition, batch‐to‐batch quali‐quantitative chemical analysis, which is the quantification of the EO volatiles to guarantee their quality and their required composition in the transformation process, should be required in each intervention session in order to check the chemical stability of the tested EO sample before its administration. Furthermore, the carrier oil used in the aromatic sample preparation should be described to study the stability and the correct EO posology in each exposure experimental session.

Regarding diffusion systems, EO stability should be guaranteed under controlled temperature and sample oxygen exposure in order to avoid any sort of EO degradation. Considering the direct or indirect administration systems during the exposure to EO, an oxygen mask with controlled EO flow and soaked cotton pad could be considered the most effective method to reduce this problem as compared to room diffusion through candles or electric systems. However, the lab‐scale oxygen mask is generally described without any information about the operative flow. In this case, it is extremely important to focus on the composition of EO and the vehicle suitability to adjust the right posology but also to avoid any allergic or undesirable reaction. For this reason, in the case of facial masks, more than in other types of diffusion systems, different doses of lavender EO should be administered in order to shed light on the minimum quantity needed to induce an effect on cognition. Although facial masks might guarantee a better controlled EO flow in administration protocols compared to environmental diffusion, its alleged placebo effect on the subject is not to be neglected in the evaluation of lavender EO inhalation effects.

Concerning cognitive enhancing effects, the experimental heterogeneity of the selected studies did not allow conducting a meta‐analysis investigation. However, in the light of the foregoing evidence, we can suggest that aromatherapy might be considered a burgeoning practical and non‐invasive method to enhance cognitive functions, for example to prevent work‐related lapses of cognitive performance, such as car accidents, medication errors, diagnosis failure (Hanowski et al., 2009; Nichols et al., 2008). Stronger conclusions can be obtained with the adoption of more specific and accurate experimental protocols for EO inhalation, since NOS for quality assessment revealed adequate but low scores, due to lack of information provided with regard to EO source, extraction and analyses and due to lack of control of biasing variables such as placebo or expectancy effect. In particular, the investigation of cognitive effects duration and the introduction of appropriate placebo conditions are required to better understand the underlying mechanisms of EO inhalation. To disentangle this issue, a placebo group with inert odor administration should be included and biasing contextual cues such as diffusion devices should be controlled. In this context, to avoid subject biasing and simultaneously prevent EO dispersion, treatment administration in delimited areas such as cubicles and the employment of a hidden diffusion device with a flowmeter should be considered.

Generalization of cognitive enhancing effects to clinical conditions that imply cognitive impairment, such as dementia, was not possible, since one of the eligibility criteria of the present systematic review was to include studies on healthy subjects.

## CONCLUSIONS

From this systematic review, it was not possible to determine whether different EO components ratios have different impacts on cognitive function modulation, although cognition enhancement induced by lavender EO inhalation was observed. However, the employment of different diffusion devices might have contaminated the pure effect of EO on cognition, due to EO degradation in indirect systems and expectancy effect induced by subjects' awareness of aroma inhalation in direct systems. Nevertheless, despite these biasing variables, lavender EO inhalation was shown to produce a decrease of arousal level and an improvement of sustained component of attention. Robust conclusion as regards memory domain cannot be stated, since only two studies investigated this function, reporting opposite results.

Whatever further research outcomes in the field of aromatherapy will be achieved, selecting lavender EO by its quality in order to perform a suitable posology and avoid any adverse effect will be crucial as a starting point for any experimental procedure.

## CONFLICT OF INTEREST

The authors declare no conflict of interest.

## Supporting information


**Table S1.** Prisma Checklist
**Table S2.** PICOS
**Table S3.** List of inclusive keywords
**Table S4.** Search steps
**Table S5.** List of excluding words
**Figure S1.** Results retrieved by year by using the search query “Aromatherapy” on MEDLINE database.
**Figure S2.** Results retrieved by year by using the search query “Aromatherapy AND lavender OR lavandula” on MEDLINE database.

## Data Availability

N/A
