# Supplementary material for: Lavender aromatherapy: A systematic review from essential oil quality and administration methods to cognitive enhancing effects
Source: Appl Psychol Health Well Being. 2021 Oct 5;14(2):663–90. doi: 10.1111/aphw.12310 (PMC9291879; doi:10.1111/aphw.12310)

**Supplementary files**

**Table S1. Prisma Checklist**

| **Section/topic** | **#** | **Checklist item** | **Reported on page #** |
| --- | --- | --- | --- |
| **TITLE** | | |  |
| Title | 1 | Identify the report as a systematic review, meta-analysis, or both. | p 1 |
| **ABSTRACT** | | |  |
| Structured summary | 2 | Provide a structured summary including, as applicable: background; objectives; data sources; study eligibility criteria, participants, and interventions; study appraisal and synthesis methods; results; limitations; conclusions and implications of key findings; systematic review registration number. | p 1 |
| **INTRODUCTION** | | |  |
| Rationale | 3 | Describe the rationale for the review in the context of what is already known. | pp 2,3 |
| Objectives | 4 | Provide an explicit statement of questions being addressed with reference to participants, interventions, comparisons, outcomes, and study design (PICOS). | p 3 |
| **METHODS** | | |  |
| Protocol and registration | 5 | Indicate if a review protocol exists, if and where it can be accessed (e.g., Web address), and, if available, provide registration information including registration number. | p 3 |
| Eligibility criteria | 6 | Specify study characteristics (e.g., PICOS, length of follow-up) and report characteristics (e.g., years considered, language, publication status) used as criteria for eligibility, giving rationale. | p 3 |
| Information sources | 7 | Describe all information sources (e.g., databases with dates of coverage, contact with study authors to identify additional studies) in the search and date last searched. | p 3 and Table S4 |
| Search | 8 | Present full electronic search strategy for at least one database, including any limits used, such that it could be repeated. | p 3 and Table S4 |
| Study selection | 9 | State the process for selecting studies (i.e., screening, eligibility, included in systematic review, and, if applicable, included in the meta-analysis). | p 3 |
| Data collection process | 10 | Describe method of data extraction from reports (e.g., piloted forms, independently, in duplicate) and any processes for obtaining and confirming data from investigators. | p 3 |
| Data items | 11 | List and define all variables for which data were sought (e.g., PICOS, funding sources) and any assumptions and simplifications made. | Table S2, Table S3, Table S4, Table S5 |
| Risk of bias in individual studies | 12 | Describe methods used for assessing risk of bias of individual studies (including specification of whether this was done at the study or outcome level), and how this information is to be used in any data synthesis. | Table 2 |
| Summary measures | 13 | State the principal summary measures (e.g., risk ratio, difference in means). | Table 1 |
| Synthesis of results | 14 | Describe the methods of handling data and combining results of studies, if done, including measures of consistency (e.g., I^2^) for each meta-analysis. | NA |

| **Section/topic** | **#** | **Checklist item** | **Reported on page #** |
| --- | --- | --- | --- |
| Risk of bias across studies | 15 | Specify any assessment of risk of bias that may affect the cumulative evidence (e.g., publication bias, selective reporting within studies). | Table 2 |
| Additional analyses | 16 | Describe methods of additional analyses (e.g., sensitivity or subgroup analyses, meta-regression), if done, indicating which were pre-specified. | NA |
| **RESULTS** | | |  |
| Study selection | 17 | Give numbers of studies screened, assessed for eligibility, and included in the review, with reasons for exclusions at each stage, ideally with a flow diagram. | p 4 and Figure 1 |
| Study characteristics | 18 | For each study, present characteristics for which data were extracted (e.g., study size, PICOS, follow-up period) and provide the citations. | p 4 and Table S2 |
| Risk of bias within studies | 19 | Present data on risk of bias of each study and, if available, any outcome level assessment (see item 12). | p 4 and Table 2 |
| Results of individual studies | 20 | For all outcomes considered (benefits or harms), present, for each study: (a) simple summary data for each intervention group (b) effect estimates and confidence intervals, ideally with a forest plot. | pp 5-9 and Table 1 |
| Synthesis of results | 21 | Present results of each meta-analysis done, including confidence intervals and measures of consistency. | NA |
| Risk of bias across studies | 22 | Present results of any assessment of risk of bias across studies (see Item 15). | p 4 and Table 2 |
| Additional analysis | 23 | Give results of additional analyses, if done (e.g., sensitivity or subgroup analyses, meta-regression [see Item 16]). | NA |
| **DISCUSSION** | | |  |
| Summary of evidence | 24 | Summarize the main findings including the strength of evidence for each main outcome; consider their relevance to key groups (e.g., healthcare providers, users, and policy makers). | pp 9-14 |
| Limitations | 25 | Discuss limitations at study and outcome level (e.g., risk of bias), and at review-level (e.g., incomplete retrieval of identified research, reporting bias). | pp 14,15 |
| Conclusions | 26 | Provide a general interpretation of the results in the context of other evidence, and implications for future research. | p 15 |
| **FUNDING** | | |  |
| Funding | 27 | Describe sources of funding for the systematic review and other support (e.g., supply of data); role of funders for the systematic review. | p 1 |

*From:*  Moher D, Liberati A, Tetzlaff J, Altman DG, The PRISMA Group (2009). Preferred Reporting Items for Systematic Reviews and Meta-Analyses: The PRISMA Statement. PLoS Med 6(7): e1000097. doi:10.1371/journal.pmed1000097

For more information, visit: **www.prisma-statement.org**.

**Table S2. PICOS**

| **Parameter** | **Inclusion criteria** | **Exclusion criteria** |
| --- | --- | --- |
| Participants | Healthy humans of any age | Animal models and patients |
| Interventions | Experimental procedure comprising lavender essential oil inhalation | Paradigms with method of administration different from inhalation |
| Comparisons | Participants undergoing lavender essential oil inhalation treatment vs participants undergoing treatment with essential oil of other aromatic plants or not undergoing any treatment |  |
| Outcomes | Assessment of cognitive functions after essential oil exposure using neuropsychological batteries and/or psychophysiological measures (blood pressure, electrodermal activity, electroencephalogram, heart rate, heart rate variability...); quali-quantitative essential oil chemical analyses; essential oil diffusion devices | Self-report measures |
| Study design | Between-subject; crossover; randomized controlled; placebo controlled; double-blind; single-blind design | Within-subject without randomized controlled design |

**Table S3. List of inclusive keywords**

| “abductive reasoning” OR “abstract analogy” OR “abstract knowledge” OR “acoustic” OR “acoustic coding” OR “acoustic encoding” OR “acoustic phonetic processing” OR “acoustic processing” OR “action” OR “action initiation” OR “action perception” OR “activation” OR “activation level” OR “active maintenance” OR “active recall” OR “active retrieval” OR “acuity” OR “adaptation” OR “adaptive control” OR “addiction” OR “affect perception” OR “affect recognition” OR “agency” OR “agreeableness” OR “altruism” OR “altruistic motivation” OR “amodal representation” OR “analog representation” OR “analogical encoding” OR “analogical inference” OR “analogical problem solving” OR “analogical reasoning” OR “analogical transfer” OR “analogy” OR “anchoring” OR “anhedonia” OR “animacy decision” OR “animacy perception” OR “anticipation” OR “antisocial personality” OR “anxiety” OR “apparent motion” OR “apperception” OR “appetite” OR “appetitive motivation” OR “arithmetic processing” OR “arousal” OR “articulation” OR “articulatory loop” OR “articulatory planning” OR “articulatory rehearsal” OR “assimilation” OR “association” OR “association learning” OR “associative priming” OR “attachment” OR “attended channel” OR “attended stimulus” OR “attention” OR “attention capacity” OR “attention shift” OR “attention shifting” OR “attention span” OR “attentional bias” OR “attentional blink” OR “attentional effort” OR “attentional focusing” OR “attentional resources” OR “attentional state” OR “attitude” OR “audiovisual perception” OR “audition” OR “auditory arithmetic processing” OR “auditory attention” OR “auditory coding” OR “auditory encoding” OR “auditory feedback” OR “auditory grouping” OR “auditory imagery” OR “auditory learning” OR “auditory lexical access” OR “auditory localization” OR “auditory masking” OR “auditory memory” OR “auditory perception” OR “auditory recognition” OR “auditory scene” OR “auditory scene analysis” OR “auditory sentence comprehension” OR “auditory sentence recognition” OR “auditory stream segregation” OR “auditory tone detection” OR “auditory tone discrimination” OR “auditory tone perception” OR “auditory word comprehension” OR “auditory word recognition” OR “auditory working memory” OR “autobiographical memory” OR “autobiographical recall” OR “automaticity” OR “autonoesis” OR “availability heuristic” OR “aversive learning” OR “backward chaining” OR “balance” OR “behavioral inhibition” OR “belief” OR “binocular convergence” OR “binocular depth cue” OR “binocular disparity” OR “binocular rivalry” OR “binocular vision” OR “bitterness” OR “blindsight” OR “body maintenance” OR “body orientation” OR “body representation” OR “border ownership” OR “capacity limitation” OR “case based reasoning” OR “categorical clustering” OR “categorical knowledge” OR “categorical perception” OR “categorization” OR “category based induction” OR “category learning” OR “causal inference” OR “central attention” OR “central coherence” OR “central executive” OR “centration” OR “change blindness” OR “chemonociception” OR “chromatic contrast” OR “chronesthesia” OR “chunk” OR “chunking” OR “circadian rhythm” OR “cognitive control” OR “cognitive development” OR “cognitive dissonance” OR “cognitive effort” OR “cognitive heuristic” OR “cognitive load” OR “cognitive map” OR “cognitive training” OR “color constancy” OR “color perception” OR “color recognition” OR “communication” OR “competition” OR “concept” OR “concept learning” OR “conceptual category” OR “conceptual coherence” OR “conceptual metaphor” OR “conceptual planning” OR “conceptual priming” OR “conceptual skill” OR “conceptualization” OR “conditional reasoning” OR “conduct disorder” OR “cone of confusion” OR “confidence judgment” OR “conflict adaptation effect” OR “conflict detection” OR “conjunction search” OR “connotation” OR “consciousness” OR “consolidation” OR “constancy” OR “constituent structure” OR “context” OR “context dependent” OR “context memory” OR “context representation” OR “contextual knowledge” OR “contingency learning” OR “contrastive stress” OR “conventionality” OR “convergent thinking” OR “conversation” OR “conversational skill” OR “conversational speech” OR “conversational structure” OR “coordination” OR “coproduction” OR “coreference” OR “covert attention” OR “creative cognition” OR “creative problem solving” OR “creative thinking” OR “critical period” OR “crossmodal” OR “crosstalk” OR “crowding” OR “crystallized intelligence” OR “cue dependent forgetting” OR “cue validity” OR “cueing” OR “curiosity” OR “dative shift” OR “decay of activation” OR “deception” OR “decision” OR “decision certainty” OR “decision making” OR “decision uncertainty” OR “decision under uncertainty” OR “declarative knowledge” OR “declarative memory” OR “declarative rule” OR “deductive inference” OR “deductive reasoning” OR “deep processing” OR “deep structure” OR “defensive aggression” OR “defiance” OR “deliberation” OR “delusion” OR “depth cue” OR “depth perception” OR “desire” OR “detection” OR “difference threshold” OR “diphthong” OR “discourse” OR “discourse comprehension” OR “discourse knowledge” OR “discourse planning” OR “discourse processing” OR “discourse production” OR “discrimination” OR “dispositions” OR “distraction” OR “distributed coding” OR “divergent thinking” OR “divided attention” OR “domain specificity” OR “dream” OR “dyslexia” OR “eating” OR “echoic memory” OR “echolocation” OR “economic value processing” OR “edge detection” OR “efficiency” OR “effort” OR “effort valuation” OR “effortful processing” OR “egocentric” OR “elaborative processing” OR “elaborative rehearsal” OR “embodied cognition” OR “emotion” OR “emotion perception” OR “emotion recognition” OR “emotion regulation” OR “emotional bonding” OR “emotional decision making” OR “emotional enhancement” OR “emotional expression” OR “emotional face recognition” OR “emotional intelligence” OR “emotional memory” OR “emotional mimicry” OR “emotional reappraisal” OR “emotional self-evaluation” OR “emotional suppression” OR “empathy” OR “encoding” OR “episodic buffer” OR “episodic future thinking” OR “episodic intention” OR “episodic learning” OR “episodic memory” OR “episodic planning” OR “episodic prediction” OR “episodic simulation” OR “error detection” OR “error signal” OR “error trapping” OR “excitation” OR “exogenous attention” OR “expectancy” OR “expertise” OR “explicit knowledge” OR “explicit learning” OR “explicit memory” OR “externalizing” OR “extinction” OR “extrinsic motivation” OR “face maintenance” OR “face perception” OR “face recognition” OR “facial age recognition” OR “facial attractiveness recognition” OR “facial expression” OR “facial happiness recognition” OR “facial recognition” OR “facial trustworthiness recognition” OR “false memory” OR “familiarity” OR “fatigue” OR “fear” OR “feature comparison” OR “feature detection” OR “feature extraction” OR “feature integration” OR “feature search” OR “feature-based attention” OR “feedback processing” OR “figure ground reversal” OR “figure ground segregation” OR “filtering” OR “fixation” OR “fixed action patterns” OR “fluid intelligence” OR “focus” OR “focused attention” OR “forgetting” OR “form perception” OR “framing” OR “frustration” OR “functional fixedness” OR “gaze” OR “generalization” OR “generic knowledge” OR “gestalt” OR “gestalt grouping” OR “global precedence” OR “goal” OR “goal formation” OR “goal maintenance” OR “goal management” OR “goal selection” OR “goal state” OR “grammatical encoding” OR “grapheme” OR “graphemic buffer” OR “grief” OR “guilt” OR “gustation processing” OR “gustatory learning” OR “gustatory memory” OR “gustatory perception” OR “habit” OR “habit learning” OR “habit memory” OR “hallucination” OR “happiness” OR “hedonism” OR “heuristic search” OR “high energy density food recognition” OR “hill climbing” OR “humiliation” OR “humor” OR “hyperactivity” OR “iconic memory” OR “illocutionary force” OR “imageability” OR “imagery” OR “imagination” OR “implicit knowledge” OR “implicit learning” OR “implicit memory” OR “imprinting” OR “impulsivity” OR “inappropriate speech” OR “inattention” OR “inattentional blindness” OR “incidental learning” OR “incubation” OR “indignation” OR “induction” OR “inductive reasoning” OR “inference” OR “inhibition” OR “inhibition of return” OR “insight” OR “instinct” OR “instrumental conditioning” OR “instrumental learning” OR “integration” OR “intelligence” OR “intention” OR “intentional forgetting” OR “intentional learning” OR “intentionality” OR “interference” OR “interference control” OR “interference resolution” OR “intermediate-term memory” OR “internal speech” OR “internalizing” OR “interoception” OR “interoceptive representation” OR “interrogative” OR “intertemporal choice” OR “intonation” OR “intrinsic motivation” OR “introspection” OR “involuntary attention” OR “irony” OR “irritability” OR “joint attention” OR “judgment” OR “kinaesthetic representation” OR “kindness priming” OR “kinesthesia” OR “knowledge” OR “language” OR “language acquisition” OR “language comprehension” OR “language learning” OR “language processing” OR “language production” OR “lateral masking” OR “learning” OR “left finger response execution” OR “left hand response execution” OR “left toe response execution” OR “lemma” OR “lethargy” OR “lexeme” OR “lexical access” OR “lexical ambiguity” OR “lexical encoding” OR “lexical processing” OR “lexical retrieval” OR “lexicon” OR “life satisfaction” OR “limited capacity” OR “linguistic competence” OR “listening” OR “localization” OR “locomotion” OR “logic” OR “logical reasoning” OR “loneliness” OR “long-term memory” OR “long term memory” OR “loss” OR “loss anticipation” OR “loss aversion” OR “lying” OR “maintenance” OR “manipulation” OR “mathematical reasoning” OR “meaning” OR “mechanical reasoning” OR “melody” OR “memory” OR “memory acquisition” OR “memory consolidation” OR “memory decay” OR “memory retrieval” OR “memory storage” OR “memory trace” OR “mental arithmetic” OR “mental imagery” OR “mental representation” OR “mental rotation” OR “metacognition” OR “metacognitive skill” OR “meta-cognitive skill” OR “metacomprehension” OR “meta-comprehension” OR “metamemory” OR “meta-memory” OR “metaphor” OR “misattribution” OR “monetary reward prediction error” OR “monitoring” OR “mood” OR “morphological processing” OR “morphology” OR “motion aftereffect” OR “motion detection” OR “motor control” OR “motor learning” OR “motor planning” OR “motor praxis” OR “motor program” OR “motor sequence learning” OR “movement” OR “multi-sensory” OR “multisensory” OR “multisensory integration” OR “multistable perception” OR “naming” OR “narrative” OR “navigation” OR “negative emotion” OR “negative feedback processing” OR “negative priming” OR “neologism” OR “neuroplasticity” OR “nociception” OR “noesis” OR “noise sensitivity” OR “non-declarative” OR “nondeclarative knowledge” OR “nondeclarative memory” OR “novelty detection” OR “numerical comparison” OR “numerical scale judgment” OR “object categorization” OR “object centered representation” OR “object detection” OR “object maintenance” OR “object manipulation” OR “object perception” OR “object recognition” OR “object-based attention” OR “obsession” OR “oddball detection” OR “offensive aggression” OR “olfaction” OR “olfactory perception” OR “openness” OR “optical illusion” OR “orthographic lexicon” OR “orthography” OR “overt attention” OR “overt naming” OR “pain” OR “pain habituation” OR “pain sensitization” OR “paranoia” OR “paraphasia” OR “parsing” OR “passive attention” OR “past tense” OR “pattern maintenance” OR “pattern recognition” OR “pavlovian conditioning” OR “perception” OR “perceptual binding” OR “perceptual categorization” OR “perceptual fluency” OR “perceptual identification” OR “perceptual learning” OR “perceptual priming” OR “perceptual similarity” OR “perceptual skill” OR “perfectionism” OR “performance monitoring” OR “phonation” OR “phonemic paraphasia” OR “phonetics” OR “phonological assembly” OR “phonological awareness” OR “phonological buffer” OR “phonological code” OR “phonological comparison” OR “phonological encoding” OR “phonological loop” OR “phonological processing” OR “phonological retrieval” OR “phonological working memory” OR “phototransduction” OR “place maintenance” OR “planning” OR “positive feedback processing” OR “positive priming” OR “potential monetary loss” OR “potential monetary reward” OR “pragmatic inference” OR “pragmatic knowledge” OR “pragmatic reasoning” OR “pre-attentive” OR “preattentive processing” OR “preconscious perception” OR “prejudice” OR “primary memory” OR “priming” OR “proactive control” OR “proactive interference” OR “problem solving” OR “procedural knowledge” OR “procedural learning” OR “procedural memory” OR “procedural rule” OR “processing capacity” OR “processing speed” OR “processing stage” OR “production of non-facial communication” OR “productive facial communication” OR “pronunciation” OR “proper noun” OR “proprioception” OR “prosodic stress” OR “prosody” OR “prospection” OR “prospective memory” OR “prospective planning” OR “prototype” OR “psychological refractory period” OR “psychosis” OR “punishment processing” OR “quantitative skill” OR “reading” OR “reasoning” OR “recall” OR “reception of facial communication” OR “reception of non-facial communication” OR “recognition” OR “reconsolidation” OR “regret” OR “rehearsal” OR “rehearsal loop” OR “reinforcement learning” OR “reinstatement” OR “relational comparison” OR “relational learning” OR “remote memory” OR “repetition priming” OR “repressed memory” OR “resistance to distractor interference” OR “resource” OR “resource limit” OR “resource sharing” OR “response bias” OR “response conflict” OR “response execution” OR “response inhibition” OR “response priming” OR “response selection” OR “restricted behavior” OR “retention” OR “retrieval” OR “retrieval cue” OR “retroactive interference” OR “reward anticipation” OR “reward learning” OR “reward processing” OR “reward valuation” OR “rhythm” OR “right finger response execution” OR “right hand response execution” OR “right toe response execution” OR “rigidity” OR “risk” OR “risk aversion” OR “risk processing” OR “risk seeking” OR “route knowledge” OR “routine” OR “rule” OR “rule learning” OR “sadness” OR “salience” OR “schema” OR “search” OR “selective attention” OR “selective control” OR “self control” OR “self knowledge” OR “self monitoring” OR “self talk” OR “semantic categorization” OR “semantic category” OR “semantic information” OR “semantic knowledge” OR “semantic memory” OR “semantic network” OR “semantic priming” OR “semantic processing” OR “semantic working memory” OR “sense of body ownership” OR “sense of ownership” OR “sensitivity to change” OR “sensory defensiveness” OR “sensory memory” OR “sentence comprehension” OR “sentence processing” OR “sentence production” OR “sentence recognition” OR “sequence learning” OR “serial learning” OR “serial processing” OR “serial search” OR “set shifting” OR “shallow processing” OR “shame” OR “shape recognition” OR “short-term memory” OR “short term memory” OR “skepticism” OR “skill” OR “skill acquisition” OR “sleep” OR “social cognition” OR “social context” OR “social inference” OR “social intelligence” OR “social motivation” OR “social norm processing” OR “social phobia” OR somatosensation” OR “sound perception” OR “source memory” OR source monitoring” OR “spatial ability” OR “spatial attention” OR “spatial cognition” OR “spatial localization” OR “spatial memory” OR “spatial selective attention” OR “spatial working memory” OR “speech perception” OR “speech processing” OR “speech production” OR “spontaneous recovery” OR “spreading activation” OR “stereopsis” OR “stereotypes” OR “stimulus detection” OR “story comprehension” OR “strategy” OR “strength” OR “stress” OR “string maintenance” OR “subconscious” OR “subjective food value” OR “subjective value judgment” OR “sublexical route” OR “subliminal perception” OR “suicidal ideation” OR “supervisory attentional system” OR “surface dyslexia” OR “surprise” OR “sustained attention” OR “synchrony perception” OR “syntactic parsing” OR “syntactic processing” OR “syntax” OR “tactile working memory” OR “task difficulty” OR “task set” OR “task switching” OR “taste aversion” OR “test term” OR “text comprehension” OR “text processing” OR “theory of mind” OR “thermosensation” OR “thought” OR “tone recognition” OR “tongue response execution” OR “tool maintenance” OR “top down processing” OR “top-down processing” OR “trait anxiety” OR “transduction” OR “traumatic memory” OR “uncertainty” OR “unconscious perception” OR “unconscious process” OR “understanding mental states” OR “unisensory” OR “updating” OR “utility” OR “valence” OR “vection” OR “verbal fluency” OR “verbal memory” OR “vestibular control” OR “visual acuity” OR “visual angle” OR “visual attention” OR “visual awareness” OR “visual body recognition” OR “visual buffer” OR “visual color discrimination” OR “visual face recognition” OR “visual form discrimination” OR “visual form recognition” OR “visual imagery” OR “visual letter recognition” OR “visual localization” OR “visual masking” OR “visual memory” OR “visual number recognition” OR “visual object detection” OR “visual object maintenance” OR “visual object recognition” OR “visual orientation” OR “visual pattern recognition” OR “visual perception” OR “visual place recognition” OR “visual pseudoword recognition” OR “visual recognition” OR “visual representation” OR “visual search” OR “visual sentence recognition” OR “visual string recognition” OR “visual tool recognition” OR “visual word recognition” OR “visual working memory” OR “visuospatial sketch pad” OR “visuospatial” OR “visuo-spatial” OR “vocal response execution” OR “voice perception” OR “wisdom” “word comprehension” OR “word generation” OR “word maintenance” OR “word order” OR “word pronunciation” OR “word recognition” OR “word repetition” OR “working memory” OR “working memory maintenance” OR “working memory retrieval” OR “working memory storage” OR “working memory updating” OR “worldview”. |
| --- |

**Table S4. Search steps**

| **Database** | **Step** | **Query** | **Research in** |
| --- | --- | --- | --- |
| MEDLINE  ERIC;  Google Scholar;  Scopus;  PsycInfo | #1 | “lavender” OR “lavandula” | Title/Abstract/Full text |
|  | #2 | Cognitive functions list retrieved from Cognitive Atlas website | Title/Abstract/Full text |
|  | #3 | Intersect #1 AND #2 |  |
|  | #4 | Exclude reviews; case reports; commentaries; articles not published in a peer-reviewed journal; gray literature; articles not available in full-text and/or in English language |  |
|  | #5 | Limit to “Humans” |  |
|  | #6 | Exclude articles containing words related to other fields of application of lavender essential oils (agricultural, botanical and medical pathologies/treatments)  Exclude articles not related to “cold” cognitive functions studies, such as Attention, Executive/Cognitive Control, Learning and Memory |  |

**Table S5. List of excluding words**

| “pesticide”, “antifeedant”, “antimicrobial”, “antifungal”, “pollution”, “phenology”, “seedlings”, “pollination”, “antibacterial”, “bioenergy”, “mosquitoes”, “phytostabilization”, “phytoremediation”, “symbionts”, “bauxite”, “dryland”, “gallate”, “nematodes”, “calorific”, “wildfire”, “ectomycorrhizal”, “fossil”, “paleobotanical”, “chlorophyll”, “starch”, “theophylline”, “kaempferol”, “nodulation”, “chloroplast”, “primordia”, “catechin”, “antimalarial”, “chitin”, “polypeptides”, “callus”, “collagen”, “symbiotic”, “ovicidal”, “phylogeography”, “pleistocene”, “anthelmintic”, “triterpenoid”, “larvae”, “larval”, “antiviral”, “colonisation”, “proanthocyanidins”, “osmoregulation”, “aridity”, “saponins”, “methanolic”, “policosanol”, “termite”, “termiticidal”, “organogenesis”, “embryogenesis”, “fuel”, “biomass”, “polyphenolic”, “tannifeorus”, “bioflavonoid”, “lignin”, “phytosterols”, “microbicides”, “triterpenes”, “ploidy”, “tanning”, “sitosterol”, “compressible”, “fiber”, “embryogenesis”, “explants”, “coacervation”, “germination”, “fossil”, “fish”, “alkaline”, “metal”, “photoprotective”, “shrubland”, “mesocosm”, “nodulating”, “infestation”, “pollen”, “phytotoxicity”, “hemicellulose”, “colorant”, “antiplasmodial”, “malaria”, “colonies”, “dye”, “acclimation”, “calli”, “lignans”, “colonization”, “transgene”, “transgenomics”, “germination”, “seedlings”, “embryogenesis”, “defence”, “inbreeding”, “foliage”, “gibberellin”, “phylogeny”, “fertilizer”, “defoliation”, “insecticide”, “phyllosphere”, “alginate”, “antimalarial”, “antihelminthic”, “ants”, “arbuscular”, “arsenic”, “aridity”, “arsenate”, “auxins”, “beetle”, “biofuel”, “biosorption”, “butterfly”, “camouflage”, “carbohydrates”, “catechins”, “cellulose”, “chlorophyll”, “chloroform”, “coleoptera”, “ectomycorrhizal”, “eggs”, “fungi”, “galls”, “germination”, “greenhouse”, “hemiparasite”, “insects”, “insecticides”, “intercropping”, “larval”, “macrolide”, “microfungi”, “multiresidue”, “mycetoma”, “mycorrhizal”, “mycotoxins”, “nectar”, “nitroimidazoles”, “oospore”, “paleo-endemism”, “parasite”, “phloem”, “photosynthesis”, “photosynthetic”, “phosphorus”, “phytopathogens”, “phylodiversity”, “plantations”, “plastome”, “polyphenols”, “polysaccharides”, “pollutant”, “pollinosis”, “predation”, “proanthocyanidins”, “quinolones”, “rainfall”, “snails”, “spore”, “soil”, “stomata”, “stomatal”, “tannins”, “tannin”, “triterpenes”, “thrips”, “xylem”, “nanospheres”, “cuticular”, “cuticle”, “suberins”, “preharvest”, “postharvest”, “HIV”, “antidiarrheal”, “algicidal”, “gengival”, “exudates”, “tincture”, “colorectal”, “hepatocellular”, “chemosensitivity”, “decoction”, “orthodontic”, “antiangiogenic”, “angiogenic”, “endothelial”, “haematodes”, “pancreatitis”, “renal”, “yeast”, “methanol”, “ethanol”, “tuberculosis”, “antiproliferative”, “plasminogen”, “orthodontic”, “angiotensin”, “psoriasis”, “micronization”, “hypouricemic”, “climacteric”, “anticandidal”, “aortic”, “fibrillation”, “phytogeographical”, “retinopathy”, “retinal”, “cytotoxic”, “necrosis”, “coronary”, “osteoclast”, “cardiomyocytes”, “contractile”, “contractility”, “cardioprotective”, “ototoxicity”, “coagulation”, “hepatitis”, “cicatricial”, “auxin”, “pediculicidal”, “osteoporosis”, “tobacco mosaic virus”, “cartilage”, “intraocular”, “agroinfiltration”, “clavicle”, “knee”, “trichromacy”, “vaccination”, “colitis”, “chemopreventive”, “obesity”, “rheumatic”, “dysmenorrhea”, “arthritis”, “osteoarthritis”, “antisalmonella”, “dentine”, “dentin”, “antiemetic”, “erosion”, “bezoar”. |
| --- |

**Figure S1. Results retrieved by year by using the search query “Aromatherapy” on MEDLINE database.**


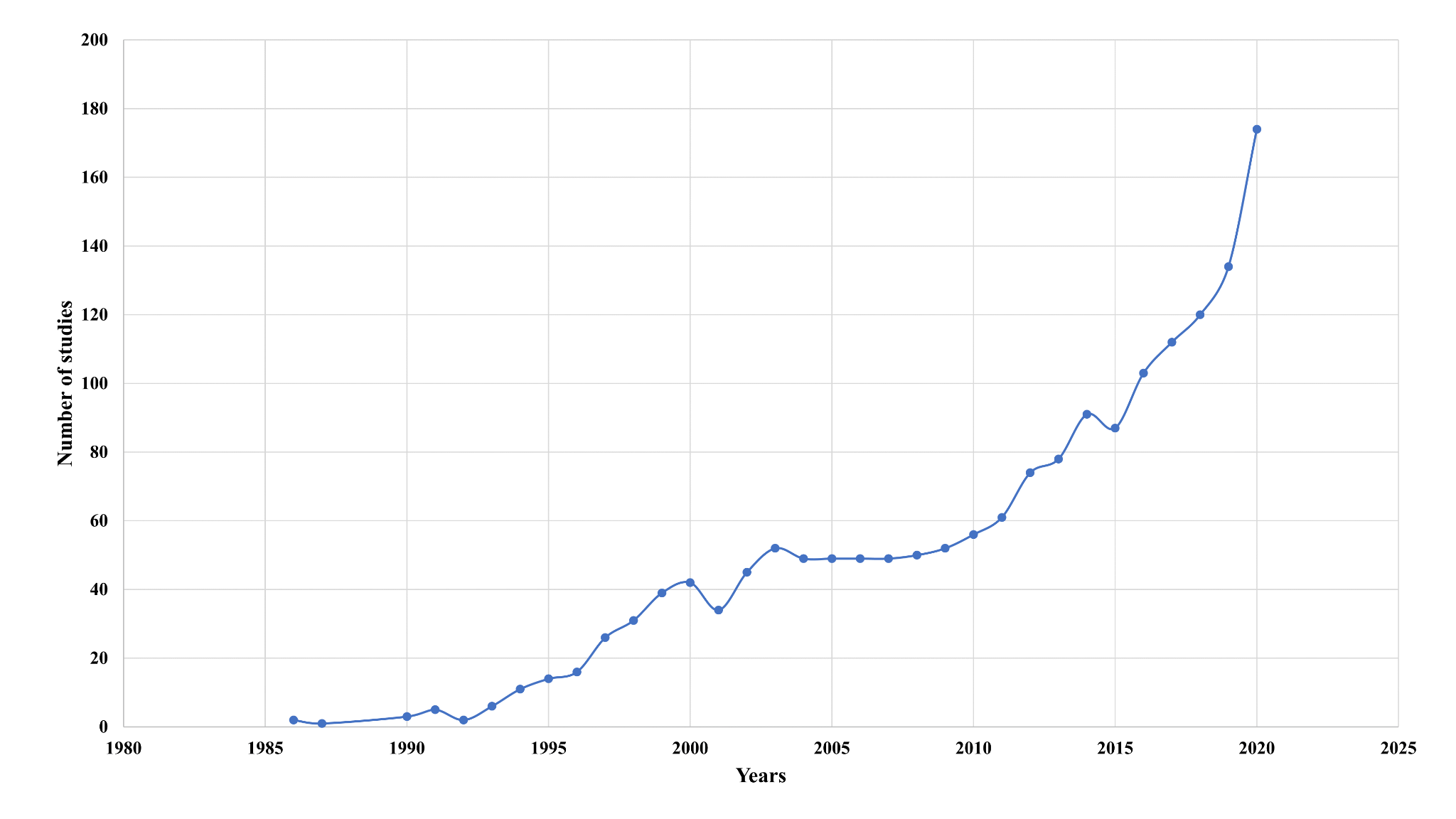


**Figure S2. Results retrieved by year by using the search query “Aromatherapy AND lavender OR lavandula” on MEDLINE database.**


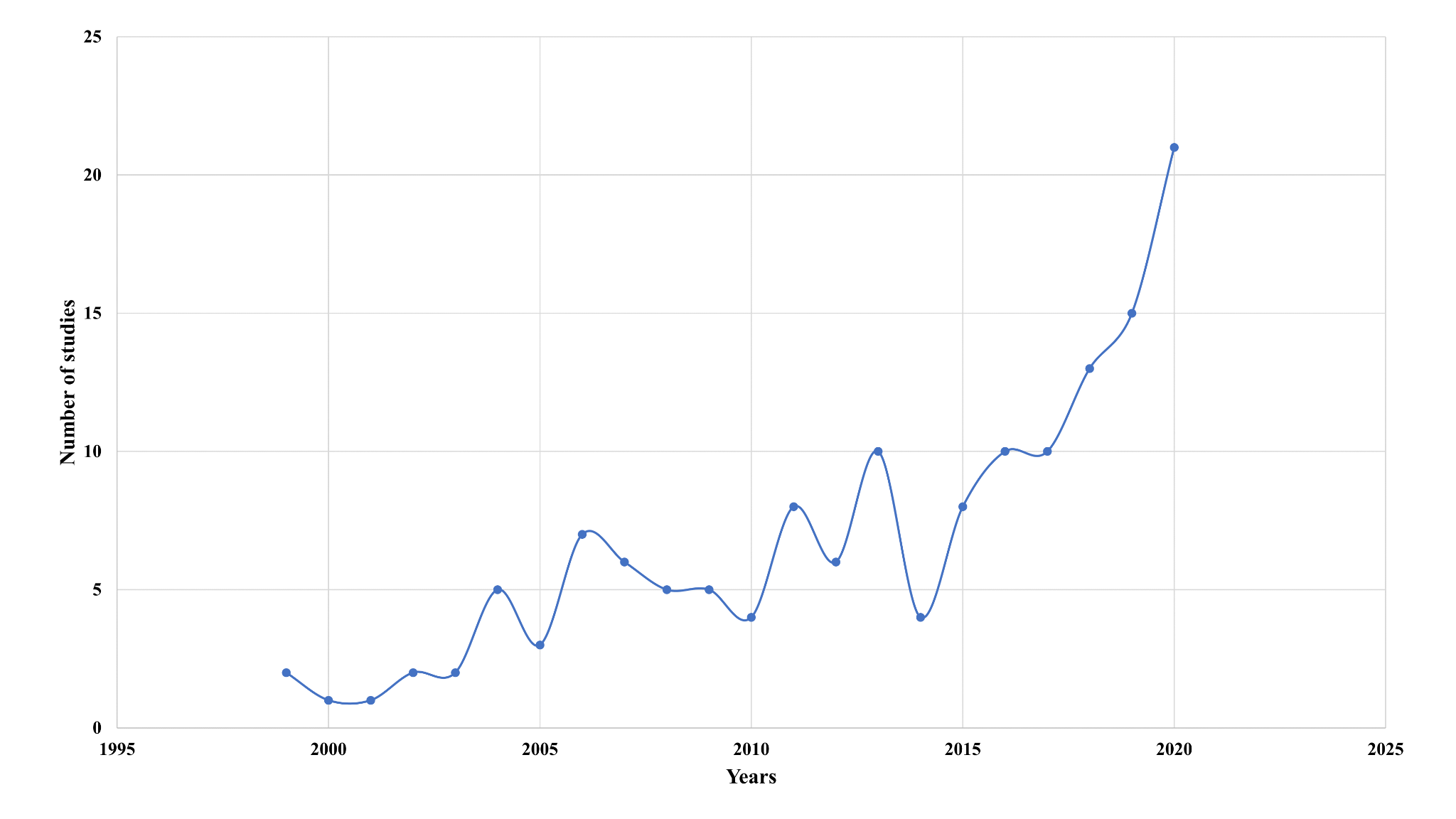

Supplement: Supplementary file 1 — Table S1. Prisma Checklist Table S2. PICOS Table S3. List of inclusive keywords Table S4. Search steps Table S5. List of excluding words Figure S1. Results retrieved by year by using the search query “Aromatherapy” on MEDLINE database. Figure S2. Results retrieved by year by using the search query “Aromatherapy AND lavender OR lavandula” on MEDLINE database. [file APHW-14-663-s001.docx]
